# Laparoscopically assisted cecal cannulation in standing horses

**DOI:** 10.3389/fvets.2025.1717140

**Published:** 2026-01-09

**Authors:** Brenda Ventura Lopes Carvalho, Maria Carolina Neves de Souza, Marcel Ferreira Bastos Avanza, Raffaella Bertoni Cavalcanti Teixeira, José Ricardo Barbosa Silva, Thiago da Silva Cardoso, Luis Gustavo e Silva Novais, Francisco Décio de Oliveira Monteiro, Rinaldo Batista Viana, Bruno Moura Monteiro, Pedro Paulo Maia Teixeira, José Dantas Ribeiro Filho

**Affiliations:** 1 Departamento de Veterinária, Universidade Federal de Viçosa (UFV), Viçosa, Minas Gerais, Brazil; 2 Instituto of Veterinary Medicine, Federal University of Pará(UFPA), Castanhal, Pará, Brazil; 3 Federal Institute of Tocantins (IFTO), Araguatins, Tocantins, Brazil; 4 Institute of Health and Animal Production, Federal Rural University of the Amazon, Belém, Pará, Brazil

**Keywords:** colic management, equine gastrointestinal tract, horse laparoscopy, typhlotomy, video-assisted surgery

## Abstract

**Introduction:**

Access to the equine cecum is required for various therapeutic and experimental procedures, including decompression, fluid therapy, and transfaunation. Traditional approaches via laparotomy are highly invasive. This study aimed to describe a minimally invasive, laparoscopically assisted technique for cecal cannulation in standing horses.

**Methods:**

Seven horses underwent the procedure under sedation and paravertebral anesthesia. Two right flank accesses were created: a 10-mm laparoscopic port for visualization and a 2-cm minilaparotomy for cecal exteriorization. A Foley catheter was inserted via typhlotomy and secured with seromuscular sutures. Postoperative management included clinical monitoring and two sessions of intracecal fluid therapy.

**Results:**

The technique was successfully completed in six of seven horses (85.7%). The catheter remained functional and was used for repeated fluid administration over 22 days without leakage. Horses maintained normal appetite, behaviour, and intestinal motility. One horse developed fatal peritonitis following immediate postoperative fluid therapy, highlighting the importance of a 24–48 h recovery period before high-volume infusion. Local wound exudation was managed effectively without systemic complications.

**Discussion:**

This standing laparoscopic technique provides a safe, practical, and minimally invasive method for establishing long-term cecal access. It offers a significant advantage over traditional laparotomy by reducing surgical trauma and enabling repeated postoperative therapeutic interventions for conditions like impaction or dysbiosis.

## 1 Introduction

Surgical access to the equine gastrointestinal tract, initially developed for experimental purposes, has become an important therapeutic tool for managing complex clinical challenges like colic (1–3, 20). Among these procedures, cecal cannulation (typhlotomy) is indicated for direct intracecal fluid therapy to address dehydration, transfaunation to restore microbial balance in cases of dysbiosis and decompression (1, 4, 5). Traditional approaches via laparotomy are highly invasive, requiring general anesthesia and carrying significant risks of postoperative pain, adhesion formation and prolonged postoperative recovery for the patient (6).

Laparoscopy has emerged as a minimally invasive alternative, reducing surgical trauma and enabling faster recovery (6, 7). The laparoscopic-assisted approach has been successfully utilized for both diagnostic and therapeutic purposes in equine gastrointestinal surgery (8, 21). It is now widely accepted as a diagnostic tool for chronic abdominal pain, particularly when performed in standing animals, and allows for the exploration of abdominal structures with minimal invasiveness (9, 10).

Surgical access to the cecum is considered when conventional therapeutic options are ineffective or unsuitable. Intravenous fluids are costly for long-term use and do not directly hydrate the cecal lumen (5, 11). Administration of fluids via a nasogastric tube is contraindicated is contraindicated in horses with reflux or gastric dysmotility (6). While percutaneous trocarization is effective for immediate, emergency cecal decompression, it is a blind, single-use procedure with inherent risks of iatrogenic injury and is not suitable for repeated access (1, 6). In contrast, traditional surgical cannulation via laparotomy provides secure, long-term access but is a major invasive procedure (6). Consequently, a minimally invasive technique for secure cecal cannulation is needed to provide a viable route for long-term enteral therapy, while avoiding the morbidity of a full laparotomy (4, 6, 12).

Cecal cannulation by video-assisted laparoscopy allows for controlled, visually-guided access to the intestinal lumen, increasing the diagnostic and therapeutic potential of the technique (7, 13). Recent studies have demonstrated the feasibility and safety of video-assisted cannulation of other visceral organs, such as the rumen and abomasum, in large animals, supporting the development of similar minimally invasive approaches for the equine cecum (14–16).

The primary clinical justification for this technique lies in the management of complex, non-acute cases where prolonged cecal access is beneficial. This includes horses with recurrent cecal impactions where repeated intracecal hydration can soften ingesta (1, 17), cases requiring long-term enteral fluid support where intravenous therapy is cost-prohibitive and nasogastric intubation is not tolerated (5, 6), and patients needing targeted microbial replenishment via transfaunation over several days (4, 12). It is not intended as a first-line emergency procedure for acute surgical colic, but rather as a planned salvage intervention for specific refractory conditions.

Therefore, the purpose of this study was to develop and evaluate a laparoscopically assisted technique for cecal cannulation in standing horses, focusing on its practicality, safety, and clinical applicability as a minimally invasive alternative to traditional laparotomy.

## 2 Materials and methods

### 2.1 Ethical approval and animals

This study was carried out according to the recommendations of the National Council for Experimentation Control in Brazil (CONCEA). This research was approved by the Animal Ethics and Welfare Committee of the Federal University of Viçosa (Protocol No. 17/2015). Seven clinically healthy horses from the Federal University of Viçosa, with an average weight of 363 ± 35 kg and 5–15 years old, were selected based on physical examination, complete blood count and serum biochemistry profile. All animals were housed in individual stalls and received fresh *Pennisetum purpureum* grass, *Cynodon* hay, commercial concentrate (Proequi 13 Laminados. Guabi. Brazil), mineral salt and water *ad libitum*.

### 2.2 Cecal cannulation

Horses were fasted for food and water were withheld for 12 and 4 h, respectively. Peri-operative antimicrobial treatment was initiated approximately 60 min before the first incision and consisted of intravenous administration of penicillin G procaine and benzathine (Penfort® PPU, Ourofino Saúde Animal, São Paulo, Brazil) at a dose of 30 mg/kg and intravenous administration of 4% gentamicin sulphate (Gentomicin 4%, Syntec, São Paulo, Brazil) at a dose of 6.6 mg/kg.

The right flank was clipped and aseptically prepared using an antiseptic based on 2% chlorhexidine digluconate (Riohex®, Rioquímica, São José do Rio Preto, São Paulo, Brazil). Proximal paravertebral blocks of the dorsal branches of spinal nerves T18, L1, L2, and L3 were performed by inserting a spinal needle immediately cranial to the transverse process of each vertebra and depositing 5 mL of 2% lidocaine subcutaneously. Distal paravertebral blocks targeting the ventral branches were achieved by advancing the needle through the intertransverse ligament and depositing an additional 5 mL of local anesthetic (Lidovet, Laboratório Bravet Ltda, Rio de Janeiro, Brazil).

Sedation and analgesia were provided by an intravenous injection of 1% detomidine hydrochloride (Detomidin®, Syntec, Santana de Parnaíba, São Paulo, Brazil) at a dose of 0.02 mg/kg and 1% butorphanol tartrate (Butorfin®, Vetnil, Louveira, São Paulo, Brazil) at a dose of 0.1 mg/kg. A continuous rate infusion of detomidine was maintained to provide a stable plane of sedation for the duration of the procedure (22). Preemptive analgesia was enhanced with intravenous administration of 8% flunixin meglumine (Flunixil®, MSD Saúde Animal, São Paulo, Brazil) at a dose of 1.1 mg/kg.

The procedure utilized a 10-mm laparoscope with a trocar and cannula (Karl Storz) for abdominal visualization in addition to standard surgical instruments, including Foerster forceps for cecal manipulation. Two right flank accesses were created: a 10-mm laparoscopic port for CO₂ insufflation (10 mmHg) and a 2-cm minilaparotomy for cecal exteriorization.

**Figure 1:**
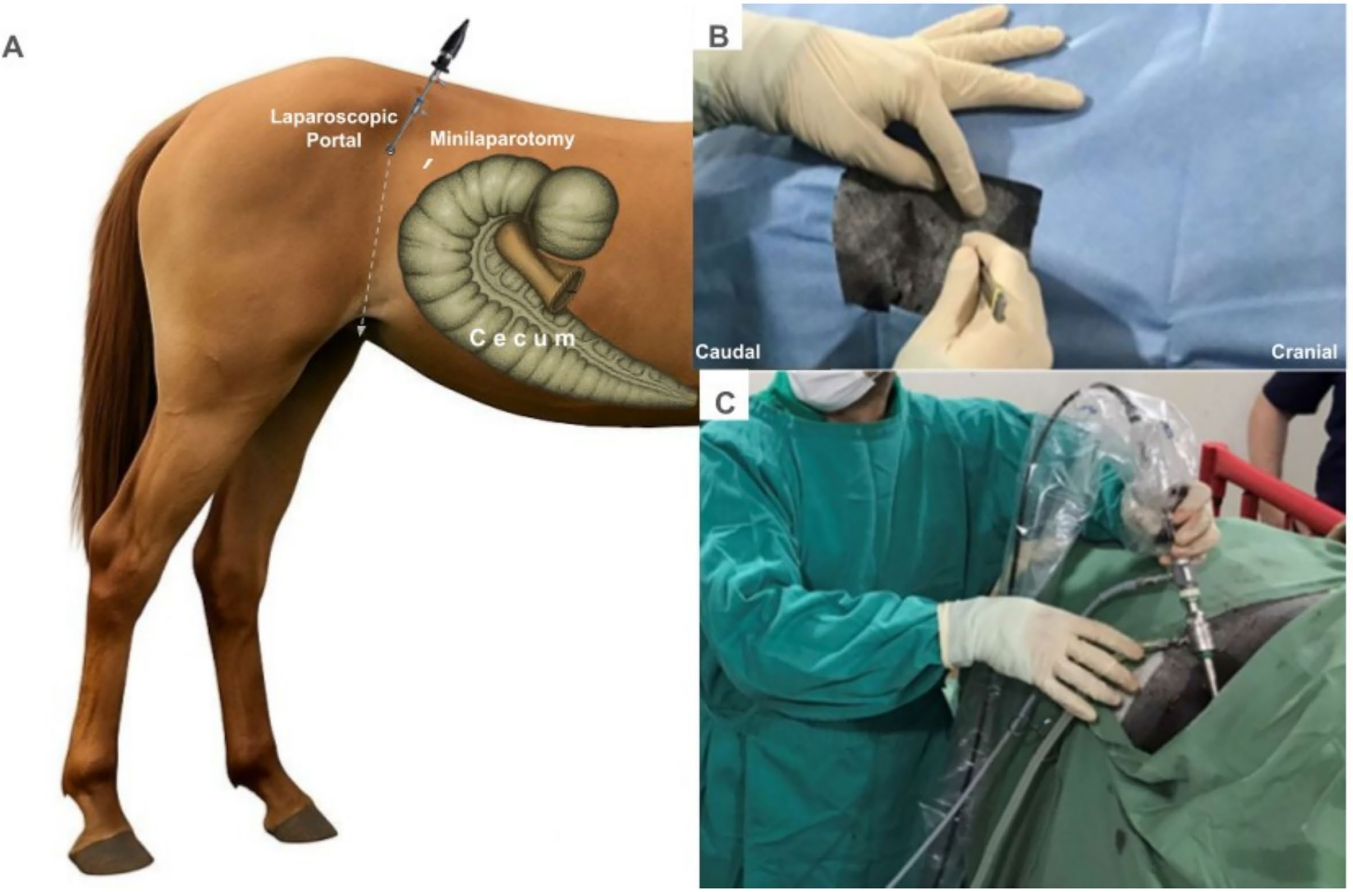
Laparoscopic-assisted approach with the establishment of the laparoscopic portal. **(A)** Position of the accesses to the abdominal cavity in relation to the equine cecum. **(B)** Incision for direct insertion of the trocar. **(C)** Establishment of the laparoscopic portal with light source and intrabdominal CO_2_ insufflation.

A laparoscopic port was established in the center of the paralumbar fossa, caudal to the last rib and ventral to the transverse processes of the lumbar vertebrae, midway between the last rib and the tuber coxae (Figure 1A). A vertical incision of approximately 10 mm was made through the skin (Figure 1B) to introduce a 10 mm laparoscopic cannula with a blunt tip obturator through the incision in a caudomedial direction (aiming for the contralateral stifle) into the abdominal cavity, using careful alternating rotation. Subsequently, the obturator was removed, and a laparoscope was inserted through the cannula for intra-abdominal visualization with the coupling of the CO_2_ insufflation hose, through which a pressure pneumoperitoneum of 10 mmHg was established with a flow speed of 5 L/min (Figure 1C).

**Figure 2:**
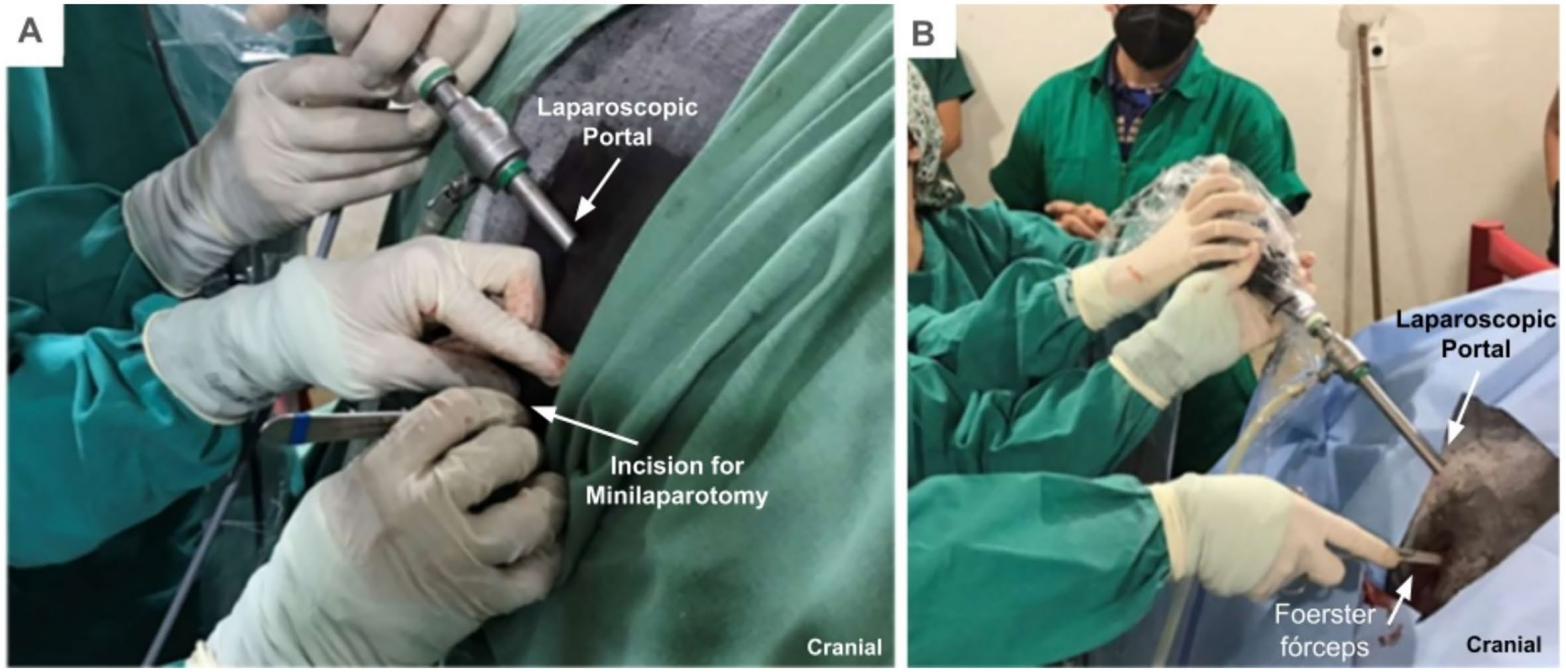
Minilaparotomy and traction of the wall of the base of the cecum. **(A)** Incision for the minilaparotmy. **(B)** Insertion of the Foerster forceps to exert traction on the wall of the base of the cecum.

A 10 mm diameter, 0-degree laparoscope was introduced into the abdomen through the first portal to identify the cecum. A skin incision measuring approximately 2 cm was made cranioventrally to the laparoscopic portal, immediately caudal to the last rib, and subsequently a blunt dissection of the subcutaneous and muscular layers was performed to access the abdominal cavity through this minilaparotomy (Figure 2A). After laparoscopic visualizing of the serosal wall of the base of the cecum, Foerster forceps were inserted through the minilaparotomy (Figure 2B). These forceps were used for the manipulation and traction of the visceral wall of the base of the cecum.

**Figure 3:**
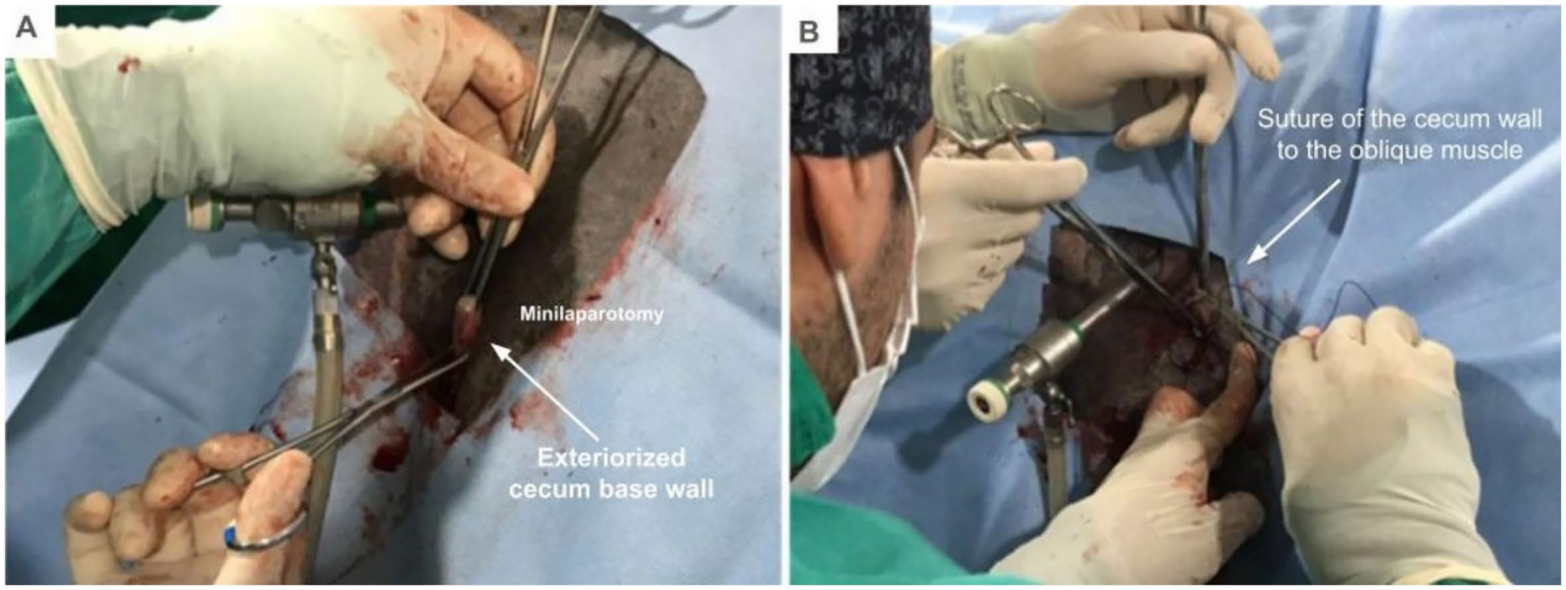
Exposure and extrabdominal suturing of the seromuscular layer of the base of the cecum to the abdominal wall. **(A)** Cecum exposed and held in place by two Allis forceps. **(B)** Construction of anchor suture points.

With the Foester forceps, the wall of the cecum was pulled and exteriorized from the abdominal cavity, being held in place by two Allis forceps (Figure 3A). The part chosen for suture was lateral, close to the base and to the greater curvature, avoiding the taenia. Four anchor suture points were made at the 3, 6, 9, and 12 o’clock positions using absorbable USP size-0 poliglecaprone suture (Figure 3B).

**Figure 4:**
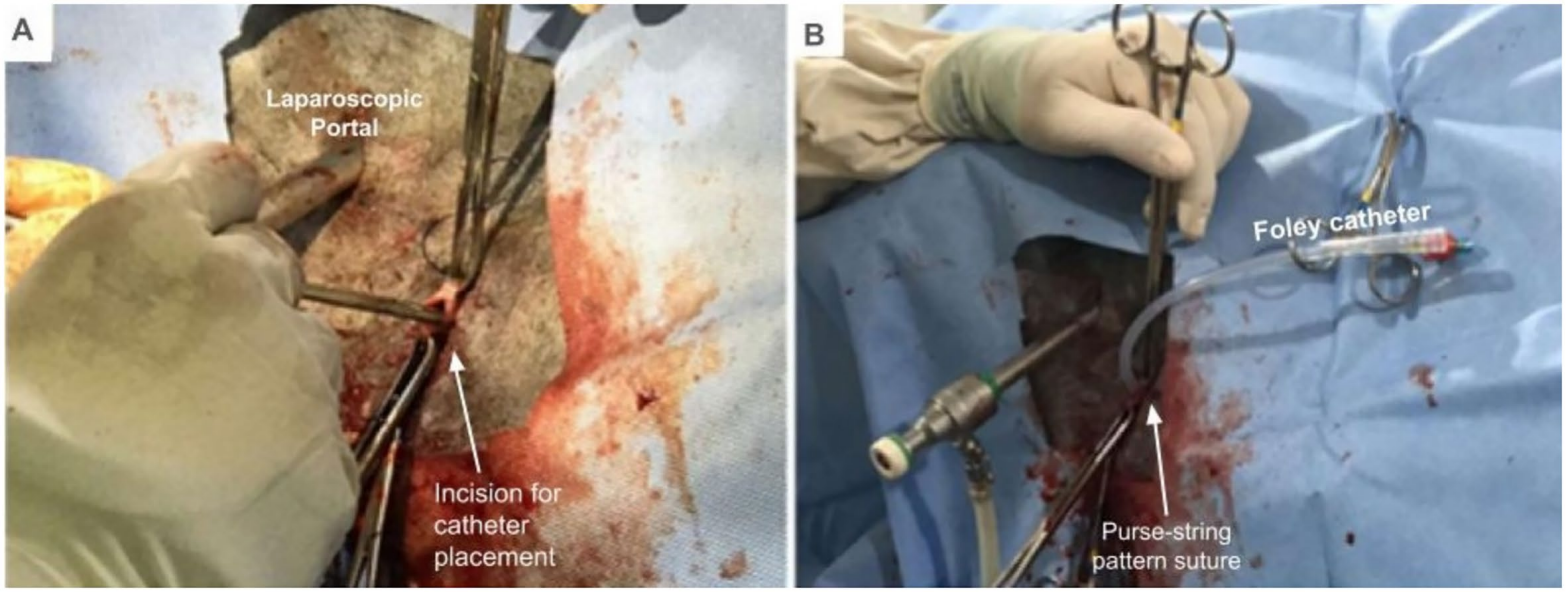
Catheter placement by typhlotomy. **(A)** Puncture incision of cecum. **(B)** Insertion of the foley catheter.

After suturing the seromuscular layer of the cecum to the external abdominal oblique muscle, a stab incision was made in the cecum and a silicone foley catheter 18F was inserted (Figures 4A,B). A pursestring pattern suture was placed in the skin around the foley catheter for definitive fixation.

**Figure 5:**
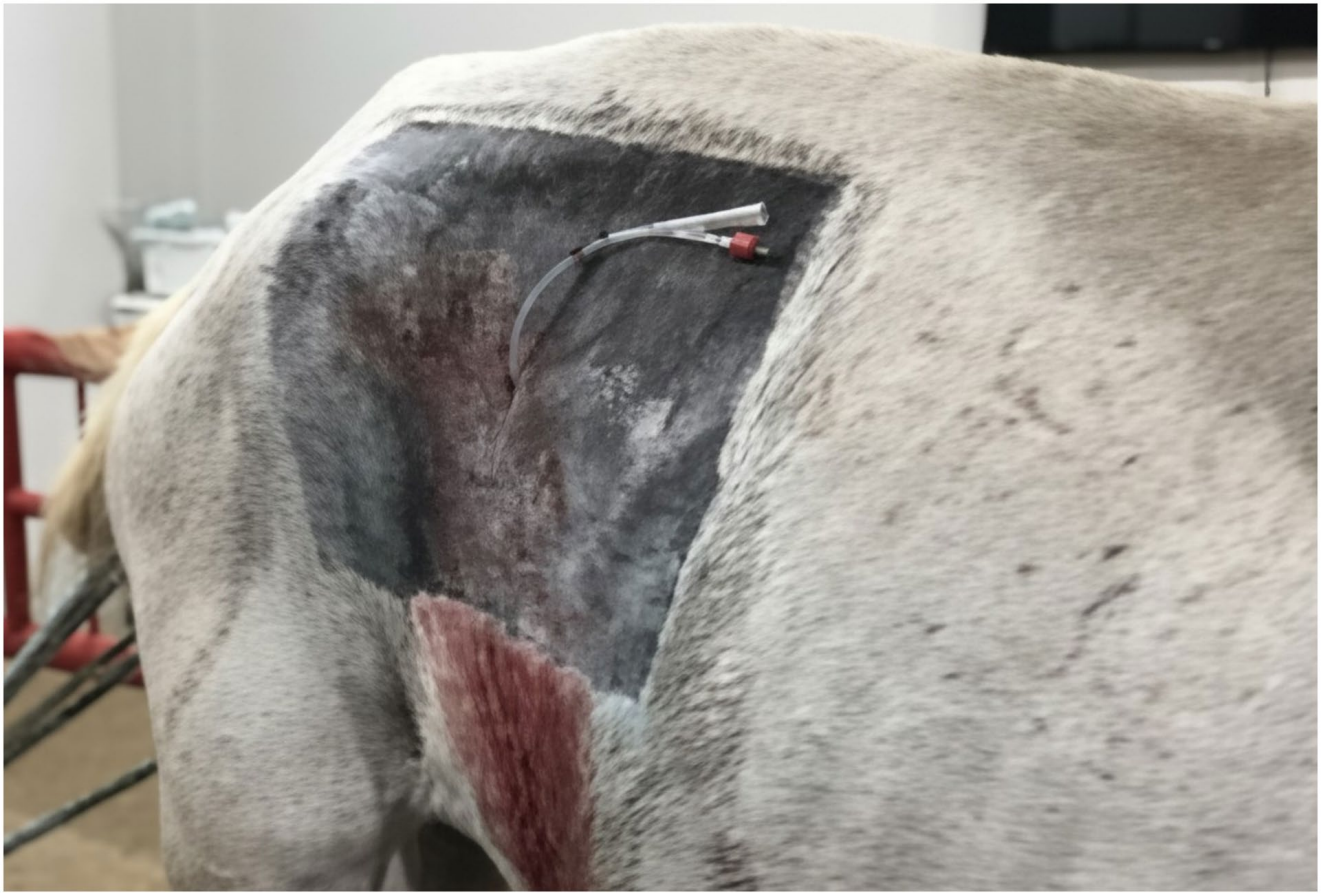
Cecal cannulation in standing horses, skin suture for catheter fixation and finalized procedure.

The laparoscopic portal was closed with an interrupted intradermal suture and the same suture was used to close the incisional space on the skin above and below the foley catheter (Figure 5).

The balloon in the catheter was filled with 20 mL of sterile 0.9% sodium chloride solution. To provide further stability and prevent dangling, the catheter tip was secured to the skin with a separate suture (Figure 5). The site was then cleaned with sodium chloride 0.9% and chlorhexidine and a sterile gauze compress was placed over, leaving only the catheter tip exposed.

Postoperative analgesia consisted intramuscular administration of flunixin meglumine (Flunixil®, MSD Saúde Animal, São Paulo, Brazil) at a dose of 1.1 mg/kg administered every 12 h for 48 h, followed by dose of 1.1 mg/kg every 24 h for an additional 3 days and post operative antimicrobial treatment consisted of intramuscular administration of G penicillin (Penfort® PPU, Ourofino Saúde Animal, São Paulo, Brazil) at a dose of 30 mg/kg every 24 h, initiated 30–60 min prior to surgery and continued for 3 days postoperatively.

### 2.3 Post-surgical evaluation

The animals were evaluated every 6 h for the first 48 h and then twice daily for the following week. Thereafter, all horses received a daily clinical examination until the end of the study on day 22. Assessments included behavioural observations related to attitude, appetite, response to human interaction. The assessment of signs of colic (looking at the flank, pawing, rolling); physiological parameters such as heart rate, respiratory rate, rectal temperature and clinical signs such as presence of wound sensitivity (e.g., heat, swelling and pain), intestinal motility through auscultation, and posture.

Physical examination included monitoring of heart rate (beats per minute), respiratory rate (breaths per minute), rectal temperature (°C), and intestinal motility via auscultation. The abdomen was divided into four quadrants (left/right, dorsal/ventral), and after 1 min of auscultation per quadrant, motility was scored on a scale of 1 (absent) to 3 (vigorous). Mucose membranes of the mouth assessment evaluated color and moisture each on a 3-point scale for color, 1 (pale), 2 (normal), 3 (red) and for moisture, 1 (dry), 2 (tacky), 3 (normal). Capillary refill time (seconds) was also recorded. Thoracic and abdominal circumference were measured.

Faeces samples were collected after spontaneous defecation to determine moisture content. The samples were weighed immediately to obtain the wet weight and then placed in an oven at 80 °C for dehydration. They were reweighed at 12-h intervals until a stable, constant weight was achieved, indicating that all moisture had been removed (final dry weight). The fecal moisture content was then calculated as the percentage of weight lost during the dehydration process relative to the original wet weight.

**Figure 6:**
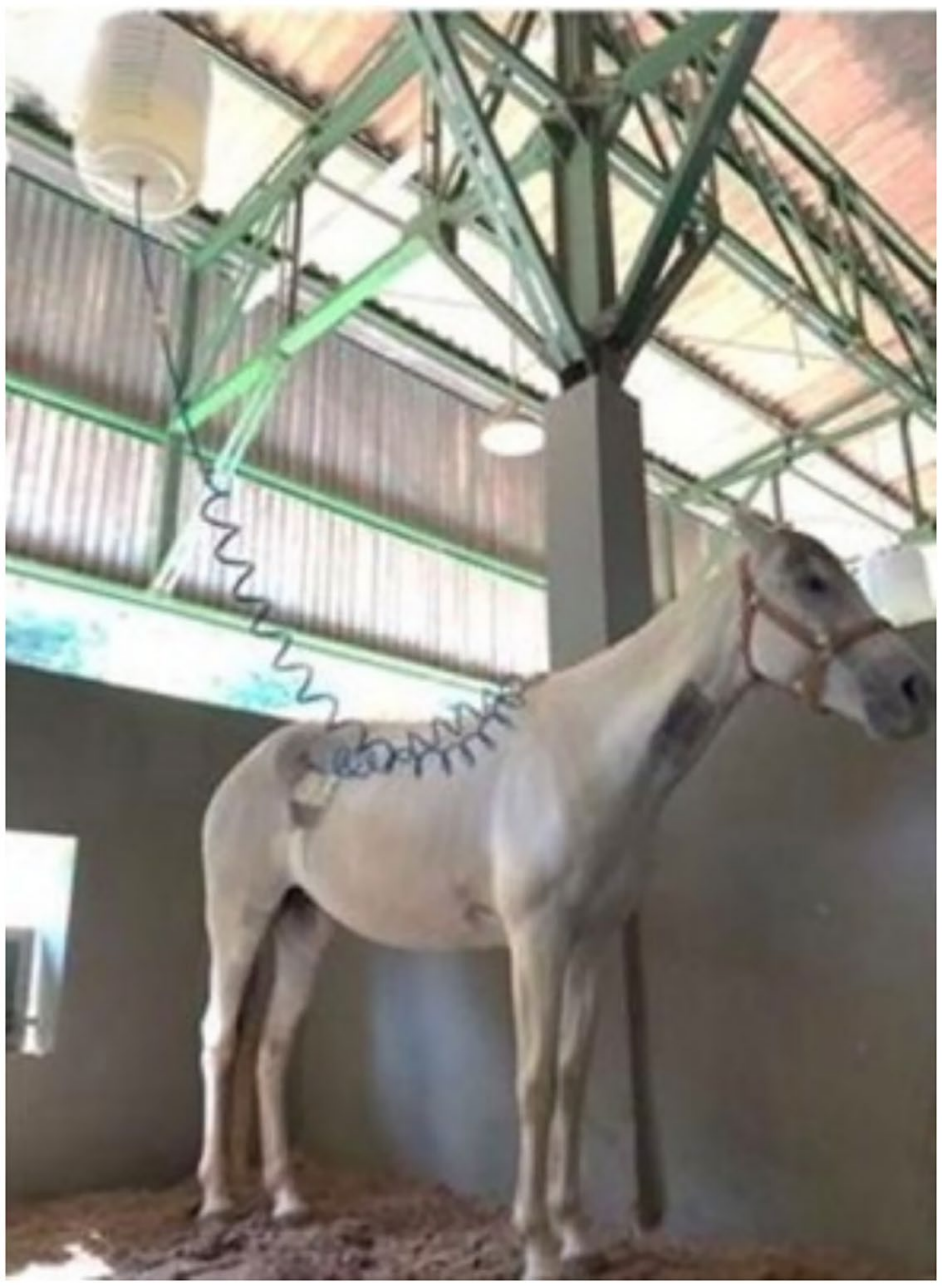
Animals receiving intracecal enteral fluid therapy.

The EFT protocol consisted of a continuous intracecal infusion of an enteral electrolyte solution (EES) at a rate of 15 mL/kg/h for 12 h. The EES composition per liter was 4.5 g sodium chloride, 0.5 g potassium chloride, 1 g calcium acetate, 0.2 g magnesium chloride, and 5 g dextrose, with a measured osmolarity of 238 mOsm L^−1^ (24). The solution was administered via the Foley catheter (100% silicone No. 18, Well Lead Medical Co.) using a closed infusion system consisting of a 20-liter reservoir and a 5-meter infusion set with a drip chamber and flow regulator (Figure 6). Between EFT sessions, the catheter was sealed with a dedicated catheter cap to prevent leakage and air entry.

Postoperative enteral fluid therapy (EFT) was administered to evaluate the functionality of the cecal catheter. For six horses, the first EFT session was conducted after a 24-h postoperative recovery period. One horse (Horse 6) received EFT immediately after surgery. A second EFT session was performed 7 days after the first for all animals.

Before each EFT session, horses were fasted for 24 h. Food and water remained withheld during the 12-h EFT infusion, resulting in a total fasting period of 36 h per session. After the 12-h infusion period, food and water were provided *ad libitum*.

### 2.4 Laboratorial analysis

Blood samples were collected by jugular venipuncture. Samples were distributed into vacuum tubes containing EDTA for complete blood count, sodium fluoride for plasma biochemistry, and clot activator for serum biochemistry. Automatic cell count was performed using the HematoClin 2.8 Vet machine (BioClin Quibasa Ltd.). A blood smear was analyzed under the microscope for differential leukocyte count. Serum osmolarity was determined by an Osmometer Model 3,320 (Advanced Instruments Inc.). Biochemical analysis was performed with BioClin 2,200 (BioClin Quibasa Ltda.) and HumaStar 300 SR (Human©).

The variables sodium, potassium, chloride, calcium, magnesium, phosphorus, fibrinogen, urea, creatinine, and total proteins were measured in serum. Lactate and glucose were measured in plasma. Acid base balance and blood gas analysis were assessed by ABL80 Flex (Radiometer Medical ApS©), and the variables used were blood pH, pCO_2_, cHCO_3_^−^, base excess (BE). The anions Gap (AG) and Strong Ion Difference (SID) were calculated from serum and urine electrolyte concentrations measured in samples collected by spontaneous urination. Containers with a capacity of 1 liter were used and an aliquot of 20 mL was taken for analysis. Urinary pH was assessed with DLA-PH (Del Lab®), urinary specific gravity was determined using a refractometer model ATC RTP-12. After these processes, the sample was centrifuged to perform biochemical analysis of urea, creatinine, sodium, potassium, chloride, calcium, magnesium, and phosphorus.

### 2.5 Statistical analysis

Quantitative data were tested for normality and homoscedasticity. The data were analyzed using analysis of variance (ANOVA) for repeated measures to compare the effects of the experimental phases (timepoints). The Tukey test was used for post-hoc comparison of means. The statistical analyses were performed using SAS software (SAS/STAT, SAS Institute Inc., Cary, NC, USA, version 9.3), and a significance level of *p* < 0.05 was adopted for all tests.

## 3 Results

The sedation and regional anesthetic protocols were effective in all seven horses, with no signs of pain or discomfort observed during the procedure. All animals recovered from sedation uneventfully. The standing position combined with a 10-mmHg pneumoperitoneum provided optimal conditions for the visualization and manipulation of the cecum. The average total surgery time was 40 min.

A 24-h fasting period effectively reduced the volume of cecum content, facilitating visualization and manipulation in six of the seven horses (85.71%). In one horse (Horse 6), normal intestinal motility was observed during laparoscopy, and its cecum contained a regular amount of ingesta at the time of incision. Despite this, the surgical procedure was completed as planned for this animal.

The surgical procedure for cecal cannulation was technically completed in all seven horses. However, long-term successful cannulation (over the 22-day study period), defined as the secure maintenance of the catheter allowing for its intended use without major complications, was achieved in six of the seven horses (85.7%). Critically, in these six animals, the intracecal catheter functioned as intended for its primary clinical purpose. One horse (Horse 6), in which the technique was technically completed and which received EFT immediately after surgery, developed fatal complications.

Clinical parameters remained stable throughout the study period in the six cannulated horses. As detailed in Table 1, heart rate, respiratory rate and rectal temperature showed no significant fluctuations from baseline values during and after intracecal fluid therapy. The intestinal motility score, while showing a transient and expected decrease after the 24-h fasting period, was maintained within a normal functional range (5–8) during the infusions and returned to baseline afterwards. Mucous membrane color and moisture scores were consistently normal, and capillary refill time remained under 2 s.

**Table 1:** Clinical and laboratory parameters (mean ± SD) in horses (*n* = 6) before and after intracecal fluid therapy sessions with an enteral electrolyte solution.

Parameter	Baseline (T-24 h)	Pre-infusion (T0 h)	4 h Infusion (T4 h)	8 h Infusion (T8 h)	12 h Infusion (T12 h)	24 h Post-infusion (T24 h)
Heart rate (bpm)	36.33 ± 5.72^A^	32.00 ± 5.66^A^	31.00 ± 4.69^A^	32.00 ± 4.38^A^	34.33 ± 3.67^A^	35.00 ± 5.02^A^
Respiratory rate (brpm)	12.80 ± 5.22^BA^	11.00 ± 3.95^BA^	11.33 ± 3.72^BA^	10.33 ± 3.67^B^	10.00 ± 4.38^B^	8.33 ± 1.97^BA^
Rectal temperature (°C)	37.38 ± 0.54^A^	36.70 ± 0.45^DC^	36.42 ± 0.44^D^	36.90 ± 0.58^DC^	37.40 ± 0.23^BAC^	37.45 ± 0.51^A^
Abdominal circumference (cm)	168.6 ± 6.02^DC^	166.1 ± 5.6^D^	171.8 ± 6.08^BDAC^	174.1 ± 5.3^BAC^	173.6 ± 5.1^BBC^	170.2 ± 4.4^BDC^
Intestinal motility (0–12)	8.00 ± 2.00^A^	5.33 ± 1.75^BC^	6.00 ± 2.28^BAC^	5.17 ± 1.83^BAC^	5.67 ± 2.66^BAC^	7.83 ± 0.41^BA^
Packed cell volume (%)	27.00 ± 2.10^BA^	24.73 ± 2.85^BA^	24.00 ± 2.83^BA^	23.86 ± 2.98^B^	24.53 ± 4.37^BA^	26.70 ± 4.31^BA^
Total protein (g/dL)	7.23 ± 0.23^A^	7.73 ± 0.35^A^	7.43 ± 0.34^A^	7.23 ± 0.43^A^	7.27 ± 0.33^A^	7.28 ± 0.18^A^
Fibrinogen (g/dL)	0.33 ± 0.10^B^	0.40 ± 0.13^BA^	0.40 ± 0.18^BA^	0.33 ± 0.10^B^	0.37 ± 0.15^BA^	0.51 ± 0.18^A^

Within a row, values with different superscript letters are significantly different (*p* < 0.05). The letters indicate statistical groupings; values that share at least one common letter are not significantly different from each other. Baseline (T-24h): 24 hours before infusion; Pre-infusion (T0h): after 24h fasting, immediately before infusion; 4h, 8h, 12h Infusion: timepoints during the 12-hour continuous intracecal fluid therapy; 24h post-infusion: 12 hours after the end of fluid therapy.

The functional efficacy of the cecal catheter for promoting luminal hydration was confirmed. Fecal moisture content, as determined by dehydration analysis, increased significantly by the end of the 12-h infusion period (T12 h), confirming effective intracecal fluid delivery and absorption (Table 2). Decrease in serum urea concentration and urine specific gravity following therapy, alongside the observed increase in fecal moisture, validating the technique’s success in achieving systemic and luminal rehydration (Table 2).

**Table 2:** Fecal moisture content (%) and urine specific gravity in horses (*n* = 6) before and after intracecal fluid therapy sessions with electrolyte solution.

Parameter	T-24 h	T0 h	T4 h	T8 h	T12 h	T24 h
Fecal moisture (%)	79.84 ± 3.38^A^	74.80 ± 2.63^BA^	75.60 ± 4.48^BA^	79.43 ± 3.01^BA^	82.20 ± 2.78^A^	79.43 ± 0.0^BA^
Serum urea (mg/dL)	25.67 ± 6.28^EDF^	33.17 ± 5.78^BAC^	33.67 ± 5.75^BAC^	30.33 ± 5.89^BDC^	26.50 ± 6.38^EDF^	26.17 ± 7.44^EDF^
Serum creatinine (mg/dL)	0.74 ± 0.22^A^	0.74 ± 0.20^A^	0.77 ± 0.24^A^	0.74 ± 0.23^A^	0.77 ± 0.23^A^	0.79 ± 0.22^A^
Urinary urea (mg/dL)	1440.9 ± 722^BA^	2146.1 ± 989^BA^	1298.2 ± 504^BA^	918.1 ± 890^BA^	131.5 ± 72^B^	922.83 ± 437^BA^
Urinary creatinine (mg/dL)	183.47 ± 93^BC^	327.27 ± 181.9^BA^	176.34 ± 100.8^BC^	76.15 ± 62.4^DC^	18.93 ± 4.9^D^	78.78 ± 41.8^DC^
Urine specific gravity	1,038 ± 16.3^A^	1,038 ± 13.6^A^	1,022 ± 11.1^BAC^	1013.5 ± 12.2^BC^	1006.3 ± 1.9^BC^	1020.4 ± 10.5^BA^

Within a row, values with different superscript letters are significantly different (*p* < 0.05). The letters indicate statistical groupings; values that share at least one common letter are not significantly different from each other. Baseline (T-24h): 24 hours before infusion; Pre-infusion (T0h): after 24h fasting, immediately before infusion; 4h, 8h, 12h Infusion: timepoints during the 12-hour continuous intracecal fluid therapy; 24h Post-infusion: 12 hours after the end of fluid therapy.

The six horses that underwent successful cannulation maintained normal appetite and behaviour throughout the 22 day postoperative monitoring period, indicating minimal systemic impact of the procedure.

**Figure 7:**
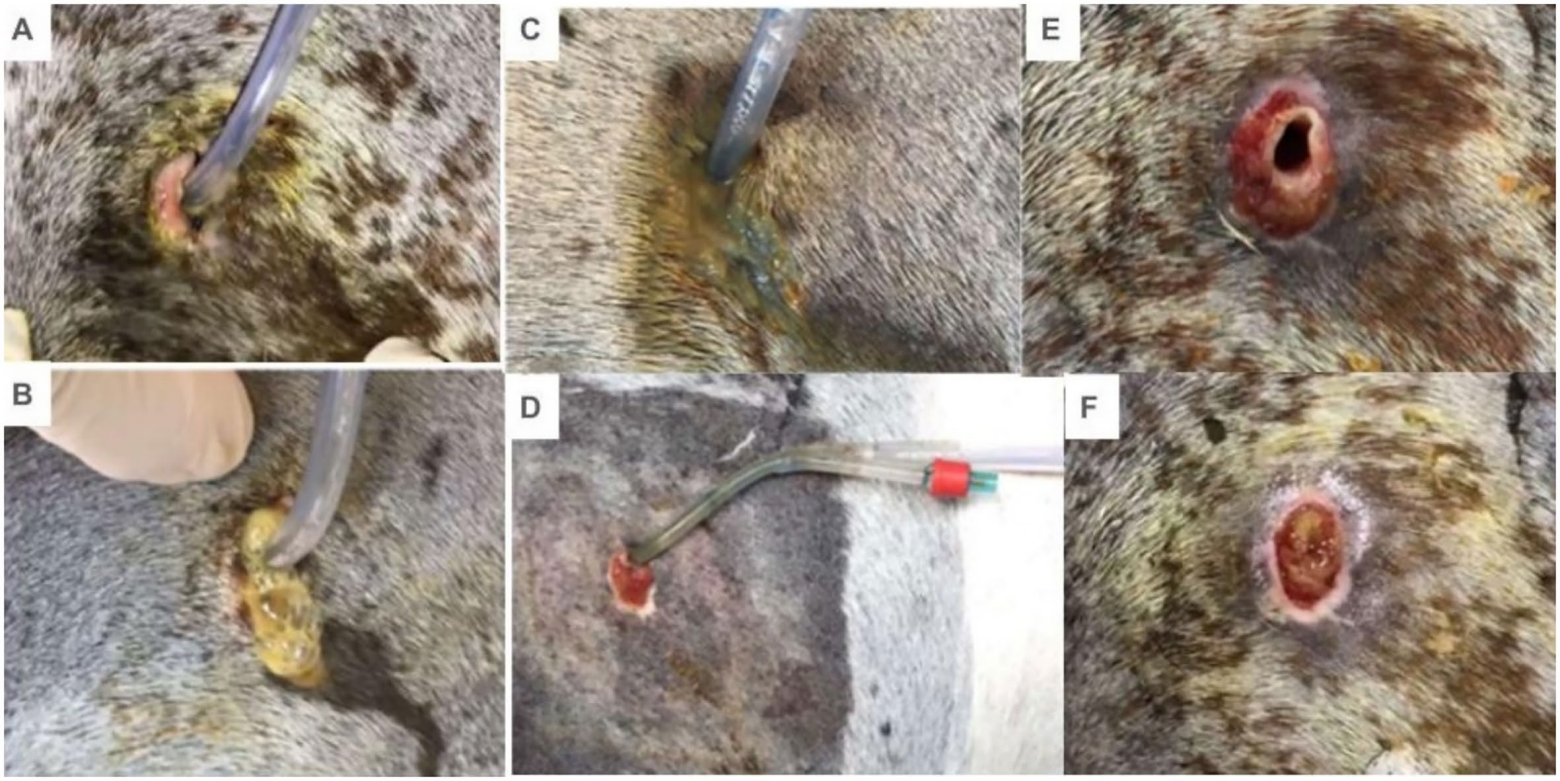
Exudation from the cecal cannulation site. **(A)** Serous exudation. **(B)** and **(C)** Purulent exudation. **(D)** The Wound healed around the catheter. **(E)** Wound immediately after catheter removal. **(F)**. Granulomatous tissue after 24 h after catheter removal.

All cannulation sites produced serous exudate in variable amounts during the first week, some progressing to a localised purulent exudate later (Figures 7A–C). Despite this, no horse showed signs of systemic infection, fever, or apathy. Local inflammation was effectively managed by a strict wound care regimen, which included daily cleansing of the cannulation site with 0.9% sodium chloride and chlorhexidine, and the application of a fresh sterile gauze compress to manage exudate and protect the area. The wounds healed satisfactorily around the indwelling Foley catheter (Figure 7D). In five horses, the catheter remained functional and securely in place for the full 22-day study period. One horse (Horse 5) self-removed its catheter on day 19, which was not replaced due to the proximity to the study’s end. Upon removal, the resulting fistula was larger than the catheter diameter (Figure 7E), and in all cases, granulomatous tissue sealed the wound within after 24 h of catheter removal (Figure 7F). All fistulas healed completely by second intention without further intervention.

**Figure 8:**
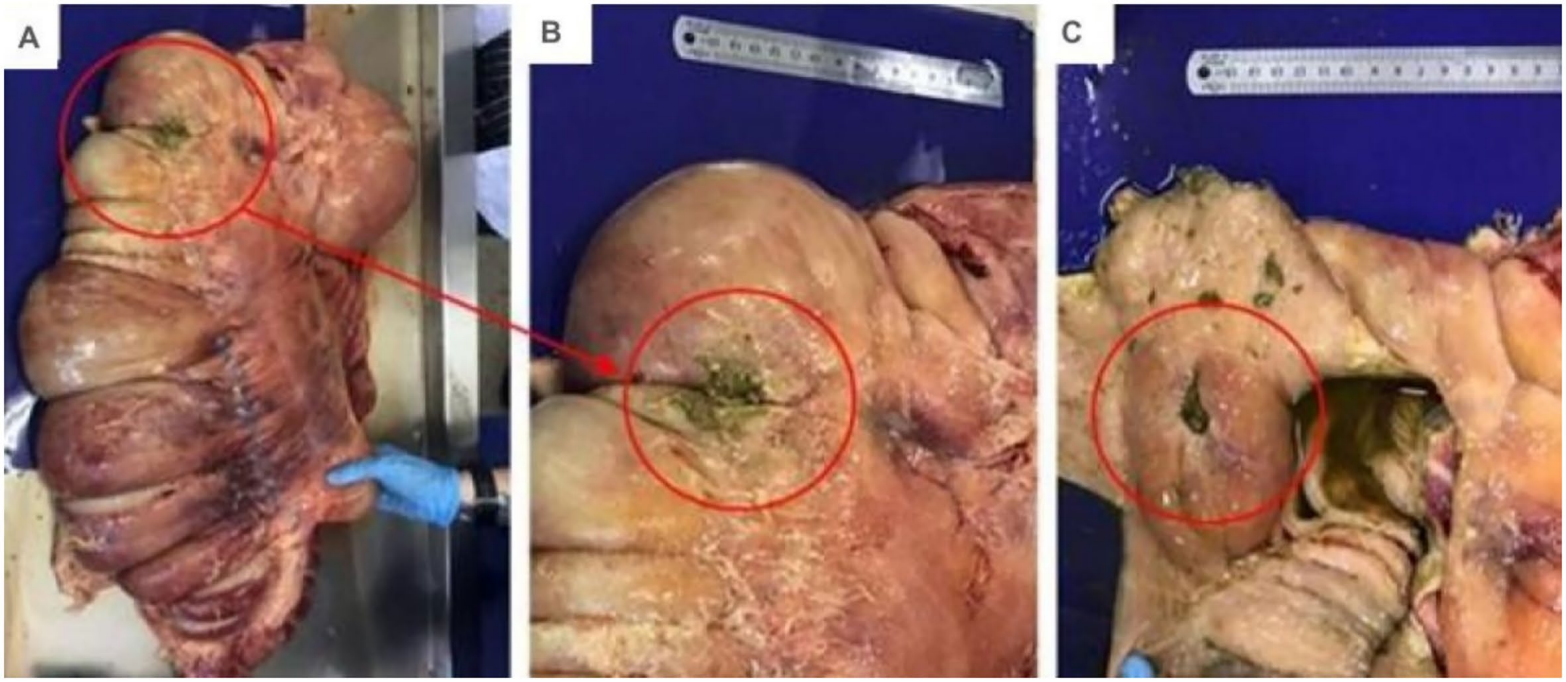
Views of the surgical wound in the cecum of horse 6, which died following postoperative complications. **(A)** General aspect of the cecum showing the location of the surgical wound in the organ after the rupture of the suture. **(B)** Closer view highlighting the area of suture dehiscence. **(C)** View of the cecal wall that shows the size of the infected surgical wound.

One horse (Horse 6), which had received intracecal fluid therapy immediately after surgery, developed fever, apathy, and anorexia on the third postoperative day. Laboratory findings progressed from leukocytosis to leukopenia with a shift to the left. The horse was removed from the experiment and put in intensive care. Treatment was started with flunixin meglumine (1.1 mg/kg intravenously once daily), ceftiofur (4.4 mg/kg intramuscular once daily), gentamicin (6.6 mg/kg intravenously once daily), metronidazole (25 mg/kg orally twice daily) and lactated Ringer’s solution (10 mL/kg/h intravenously). Despite intensive medical treatment, the horse’s condition deteriorated and the horse died as a result of post-surgical complications on day 7. Necropsy revealed peritonitis and suture dehiscence at the typhlotomy site, with no evidence of the intended adhesion between the cecum and the abdominal wall (Figures 8A–C). The exact timing of the dehiscence could not be determined.

A follow up health check 7 months after the study ended, which included a physical examination and assessment for signs of abdominal pain, confirmed that all six remaining horses were healthy and showed no signs of colic.

## 4 Discussion

This study describes and evaluates a laparoscopically assisted technique for cecal cannulation in standing horses. The procedure was successfully completed in six of seven horses, with the catheter remaining functional for 22 days, demonstrating the technique’s feasibility for establishing long-term cecal access (7, 10, 18). The single major complication, which resulted in fatal peritonitis, provides a critical caution regarding postoperative management.

The primary advantage of this technique is its minimally invasive nature, which reduces tissue trauma compared to a traditional laparotomy, a procedure associated with significant postoperative pain and adhesion formation (6). The standing position, combined with effective sedation and regional anesthesia (13, 23), provided a stable surgical field with a 10-mmHg pneumoperitoneum and allowed for a rapid recovery. The average surgery time of 40 min indicates that the procedure can be performed efficiently. These factors contributed to the minimal systemic impact observed, with all successfully cannulated horses maintaining normal appetite, behaviour, and clinical parameters throughout the study.

The technique is designed to provide a secure, long-term conduit for direct cecal access. This addresses specific limitations of conventional therapies. It offers a potential route for direct luminal hydration, which may be beneficial when long-term intravenous fluids are cost-prohibitive (5, 11, 23). Furthermore, it is a viable option when nasogastric intubation is contraindicated due to reflux (6). Unlike blind percutaneous trocarization, a single-use, emergency procedure, this method allows for controlled, initial placement under visual guidance, which may mitigate the risk of iatrogenic injury, and the indwelling catheter facilitates repeatable access (1, 6). The use of a soft silicone Foley catheter was adequate, aligning with previous reports that suggest such materials minimize mucosal irritation (13, 14, 19).

However, the technique carries inherent risks, most seriously suture dehiscence and peritonitis. The fatal complication in Horse 6, which received high-volume intracecal fluid therapy immediately postoperatively, was likely due to a mechanical overload of the fresh seromuscular sutures before a secure adhesion had formed (1, 6). While a single case cannot prove causation, it strongly suggests that immediate postoperative use of the catheter for high-volume infusion is contraindicated. We therefore recommend a 24- to 48-h recovery period to allow for initial adhesion formation before initiating fluid therapy. Local wound exudation, which occurred in all cases, was successfully managed with a consistent wound care protocol and did not lead to systemic infection in the other horses.

Our study has several important limitations. First and foremost, the technique was evaluated in healthy horses. The feasibility and safety of performing this procedure in clinical patients with pathological conditions such as severe cecal impaction or tympany remain unknown and warrant investigation (4). The fasting period used in this study to facilitate visualization and manipulation may not be feasible or safe in sick horses. Furthermore, the single occurrence of a major complication, while informative, means its exact cause and prevention cannot be definitively established.

The functional efficacy of the access was confirmed by the successful administration of intracecal fluid therapy, which resulted in a significant increase in fecal moisture content (5). This validates the catheter’s utility for promoting luminal hydration. While this establishes proof-of-concept, the therapeutic benefit of this approach for specific conditions like recurrent impactions or dysbiosis requires validation in future clinical trials (4, 12). The technique’s potential utility lies in its ability to provide a route for repeated hydration or transfaunation in complex, non-acute cases where conventional medical management has failed (1, 5). For instance, it could be considered for refractory impactions where direct hydration might soften ingesta, though the risk of rupture in a compromised cecum must be carefully considered (11, 17).

The described laparoscopically assisted cecal cannulation technique is a feasible and minimally invasive method for establishing long-term cecal access in standing horses (7, 10). Its main advantages over laparotomy include reduced surgical trauma and the potential for repeated postoperative interventions. The single major complication underscores the critical importance of a postoperative recovery period before using the catheter for high-volume infusion. While this study establishes technical feasibility in healthy horses, its clinical application for specific refractory conditions requires further investigation.

## 5 Conclusion

The described laparoscopically assisted cecal cannulation technique proved to be a relatively safe, efficient, and minimally invasive method to access the equine cecum in standing horses, with potential for application in both clinical and emergency settings. It offers advantages over traditional laparotomy, including less surgical trauma and the possibility of maintaining the cannulated cecum for complementary postoperative treatment.

## Data Availability

The raw data supporting the conclusions of this article will be made available by the authors, without undue reservation.

## References

[1] Beard WL Slough TL Gunkel CD. Technical note: a 2-stage cecal cannulation technique in standing horses. J Anim Sci. (2011) 89:2425–9. doi: 10.2527/jas.2010-3718, 21421828

[2] Firouzabadi MSS Hajikolaei MRH Baniadam A Mashhadi ARG Ghorbanpoor M. Cecal cannulation in horse; an experimental study. Iran J Vet Med. (2017) 11:353–360. doi: 10.22059/ijvm.2017.230445.1004801

[3] Silva RM Araújo LHV Cardoso TdS Franco SLI Gurgel HJ Cerqueira PHL . A single-port, multiple-access, custom-made device used in laparoscopically assisted cryptorchidectomy in standing horses - a preliminary study. Animals. (2024) 14:1091. doi: 10.3390/ani14071091, 38612330 PMC11011124

[4] Bookbinder L Prisk A. Updates on diagnosis and management of colic in the field and criteria for referral. Vet Clin North Am Equine Pract. (2023) 39:175–95. doi: 10.1016/j.cveq.2023.03.001, 37121785

[5] Souza MCN Carvalho BVL Moreira NS Marlière JP Mota JVM Avanza MFB . Intracecal fluid therapy in adult horses and use of maltodextrin as an energy source on enteral electrolyte solutions. Cienc Rural. (2025) 55:e20230556. doi: 10.1590/0103-8478cr20230556

[6] Gandini M Cerullo A Giusto G. Scoping review: occurrence and definitions of postoperative complications in equine colic surgery. Equine Vet J. (2023) 55:563–72. doi: 10.1111/evj.13881, 36199160

[7] Bertoletti A Hendrickson DA. Laparoscopic diagnostic techniques In: Ragle CA, editor. Advances in equine laparoscopy. Hoboken: Wiley (2024)Ch. 9

[8] Aitken MR. Colic surgery: recent updates. Vet Clin North Am Equine Pract. (2023) 39:249–62. doi: 10.1016/j.cveq.2023.03.009, 37169619

[9] Jones A. Standing laparoscopic exploration of the small intestines In: Ragle CA, editor. Advances in equine laparoscopy. Hoboken: Wiley (2024)Ch. 11

[10] Pudert T Cruz AM Röcken M. Use of flank laparoscopy in the standing horse as a diagnostic aid in horses with chronic abdominal pain. Equine Vet Educ. (2025) 37:299–307. doi: 10.1111/eve.14021

[11] Lawson AL Sherlock CE Ireland JL Mair TS. Equine nutrition in the post-operative colic: survey of diplomates of the American colleges of veterinary internal medicine and veterinary surgeons, and European colleges of equine internal medicine and veterinary surgeons. Equine Vet J. (2021) 53:1015–24. doi: 10.1111/evj.13381, 33174212 PMC8451781

[12] Arnold CE Pilla R. What is the microbiota and what is its role in colic? Vet Clin North Am Equine Pract. (2023) 39:381–97. doi: 10.1016/j.cveq.2023.03.004, 37121786

[13] Monteiro FDO Borges LPB Cardoso TDS Teixeira PPM Filho DZ Sartori VC . Animal model of video-assisted cecum and ileum instrumentation for equine visceral pain study. J Equine Vet Sci. (2022) 108:103799. doi: 10.1016/j.jevs.2021.103799, 34856499

[14] Santos GMA Barbosa AEC Borges LPB Morais HLM Guilherme BC Siqueira LS . Minimally invasive video-assisted rumenostomy in sheep. Small Rumin Res. (2018) 167:78–81. doi: 10.1016/j.smallrumres.2018.07.023

[15] Santos GMA Borges LPB de Morais HLM Guilherme BC Albuquerque RS Rossy KC . Percutaneous ruminostomy guided by rumenoscopy: study in an experimental model in bovine fetus. BMC Vet Res. (2022) 18:41. doi: 10.1186/s12917-022-03143-5 35039024 PMC8762941

[16] Gurgel HJ Monteiro FDO Barroso JPM Sousa LA Santos GMA Rossy KC . Laparoscopy assisted abomasal cannulation in cadavers of bovine fetuses. BMC Vet Res. (2022) 18:378. doi: 10.1186/s12917-022-03473-4 36284310 PMC9598014

[17] Gardner A Dockery A Quam V. Exploratory celiotomy in the horse secondary to acute colic: a review of indications and success rates. Top Companion Anim Med. (2019) 34:1–9. doi: 10.1053/j.tcam.2018.11.001, 30808489

[18] Jones ARE Ragle CA Anderson D Scott C. Laparoscopic evaluation of the small intestine in the standing horse: technique and effects. Vet Surg. (2017) 46:812–20. doi: 10.1111/vsu.12664, 28460413

[19] Mealey RH Carter GK Roussel AIJ Ruoff WW. Indwelling cecal catheters for fluid administration in ponies. J Vet Intern Med. (1995) 9:347–52. doi: 10.1111/j.1939-1676.1995.tb01096.x, 8531182

[20] Gillen A Archer DC. Epidemiology of colic. Vet Clin North Am Equine Pract. (2023) 39:157–74. doi: 10.1016/j.cveq.2023.03.005, 37268523

[21] Straticò P Guerri G Palozzo A Varasano V Petrizzi L. Current use of equine laparoscopy in urogenital disorders: a scoping review of the literature from 2000 to 2021. Vet Sci. (2022) 9:41. doi: 10.3390/vetsci9020041, 35202295 PMC8876348

[22] Vitoria A Romero A Fuente S Barrachina L Vazquez FJ. Application of a laparoscopic technique for vasectomy in standing horses. Vet Rec. (2019) 185:345. doi: 10.1136/vr.105396, 31409746

[23] Devick IF Leise BS Rao S Hendrickson DA. Evaluation of post-operative pain after active desufflation at completion of laparoscopy in mares undergoing ovariectomy. Can Vet J. (2018) 59:261–6. doi: 10.1177/1098612X17751923, 29599556 PMC5819046

[24] Ribeiro Filho JD Farias SK Fonseca LA Avanza MFB Dantas WMF Dias DCR . Enteral electrolyte solutions with different osmolarities: clinical and laboratory assessment in equines. J Equine Vet Sci. (2015) 35:673–8. doi: 10.1016/j.jevs.2015.06.011

